# Timing of Calorie Restriction in Mice Impacts Host Metabolic Phenotype with Correlative Changes in Gut Microbiota

**DOI:** 10.1128/mSystems.00348-19

**Published:** 2019-12-03

**Authors:** Liying Zhang, Xinhe Xue, Rui Zhai, Xin Yang, Hui Li, Liping Zhao, Chenhong Zhang

**Affiliations:** a State Key Laboratory of Microbial Metabolism, Joint International Research Laboratory of Metabolic & Developmental Sciences, School of Life Sciences and Biotechnology, Shanghai Jiao Tong University, Shanghai, China; b Department of Biochemistry and Microbiology and New Jersey Institute for Food, Nutrition and Health, School of Environmental and Biological Sciences, Rutgers University, New Brunswick, New Jersey, USA; Dalhousie University

**Keywords:** calorie restriction, shift of feeding time, gut microbiota, anti-inflammation

## Abstract

Calorie restriction (CR) is accompanied by self-imposed daily restriction of food intake and an extended fasting period between meals. The impact of restricting feeding to the dark or light phase on the effects of CR remains elusive. Here, light-fed CR mice showed physiological changes, such as muscle loss, concomitant with changes in the gut microbiota structure and composition. After switching to *ad libitum* access to food, light-fed mice had a period of food-craving behavior and short-lived physiological changes, while dark-fed mice displayed lasting changes in fat accumulation, glucose metabolism, intestinal barrier function, and systemic inflammatory markers. Moreover, the gut microbiota was modulated by when the food was consumed, and the most abundant Lactobacillus operational taxonomic unit (OTU) promoted by CR was enhanced in dark-fed mice. After switching to *ad libitum* feeding, the gut microbiota of dark-fed mice returned to the state resembling that of mice fed normal chow *ad libitum*, but that of light-fed mice was still significantly different from the other two groups. Together, these data indicate that for CR, restricting food consumption to the active phase brought better metabolic phenotype associated with potentially beneficial structural shifts in the gut microbiota.

**IMPORTANCE** Aberrant feeding patterns whereby people eat more frequently throughout the day and with a bias toward late-night eating are prevalent in society today. However, whether restriction of food to daytime in comparison to nighttime, coupled with restricted calorie intake, can influence gut microbiota, metabolism, and overall health requires further investigation. We surveyed the effects of the shift in feeding time on gut microbiota and metabolic phenotype in calorie-restricted mice and found that avoiding eating during the rest period may generate more beneficial effects in mice. This work strengthens the evidence for using “when to eat” as an intervention to improve health during calorie restriction.

## Abstract (precis)

Aberrant feeding patterns whereby people eat more frequently throughout the day and with a bias toward late-night eating are prevalent in society today. However, whether restriction of food to daytime in comparison to nighttime, coupled with restricted calorie intake, can influence gut microbiota, metabolism, and overall health requires further investigation. We surveyed the effects of the shift in feeding time on gut microbiota and metabolic phenotype in calorie-restricted mice and found that avoiding eating during the rest period may generate more beneficial effects in mice. This work strengthens the evidence for using “when to eat” as an intervention to improve health during calorie restriction.

## INTRODUCTION

Calorie restriction (CR) has been shown to extend life span and improve health in various animal models (1, 2). Long-term calorie restriction of mice, with either a normal or high-fat diet, alleviates early life metabolic phenotypes and leads to changes in liver gene expression, which could effectively reflect causal factors critical for life span-extending effects (3). Calorie-restricted mice are considered a healthier group than their *ad libitum* companions. An important way in which CR improves health during aging is through attenuation of chronic and systemic inflammation (4–6). Prior studies have reported that age-associated microbial dysbiosis aggravates intestinal permeability and systemic inflammation (7). Furthermore, results from our previous studies in mice showed that CR changed gut microbiota into a Lactobacillus-dominated structure, and a strain of Lactobacillus murinus isolated from CR mice contributed to the alleviation of aging-associated inflammation, thereby demonstrating a microbiota-dependent mechanism for the metabolic improvement by CR (8–10).

The gut microbiota also plays a pivotal role in clock-nutrition interplay (11). First, the composition of gut microbiota exhibits diurnal oscillations, which are mostly driven by quality of the diet and food consumption rhythmicity (12–14). Second, the feeding rhythm is critical, but host circadian factors also affect the microbial oscillations (15). Disruption of the host circadian clock by mutation of clock genes or by jetlag blunts the diurnal oscillation in gut microbiota composition, while timed feeding can restore microbiota oscillations in mice deficient in circadian clock genes or a functional circadian clock (12, 13, 16). Third, depletion of the microbiota disrupts circadian rhythmicity of gene expression, not only locally in the intestinal epithelium (17), but also in distal organs such as the liver (18).

It is important to note that CR is also accompanied by self-imposed feeding behavior changes, tending to cause gorging instead of slower-paced intake in mice (1, 19, 20). Seventy percent of *ad libitum* intake for the CR mice is provided once daily, which means that they have 24 h in which to consume their food allotment. However, CR mice tend to consume food within the first 2 h in which it is available, and the consequence of this behavior is fasting for 22 h until food is next available (19). Food composition (such as the proportion of fat in the diet) and food quantity (through the control of calorie intake) profoundly affect gut microbiota and health outcomes (9, 15). Moreover, an emerging area of research is the investigation of feeding time and thus changed feeding parameters, such as feeding duration and timing of food access (1, 19, 20). Aberrant time-restricted feeding may be involved in the development of disease and the acceleration of aging (21–24). In light of the mainly nocturnal feeding habits of mice, the self-imposed 2-h/22-h feeding-fasting cycles under the CR regimen and the nutrition-microbiota-clock interplay, when the food is made available (dark or light phase), may impact the benefits of CR on the animals.

In this study, to evaluate the influence of shifting feeding time on the effectiveness of CR and the gut microbiota, mice were placed on a light-fed or dark-fed CR protocol, and their metabolic phenotype and gut microbiota were investigated. The lasting effects of these two types of CR were explored by switching the mice to an *ad libitum* feeding schedule after the 4-week CR period. Light-fed CR mice displayed physiological changes such as muscle loss and changes in the structure and composition of gut microbiota. Compared to their dark-fed CR companions, light-fed mice showed a period of food-craving behavior after shifting to *ad libitum* feeding. Moreover, the gut microbiota of light-fed mice was still significantly different from dark-fed mice and the mice fed *ad libitum*, concomitant with short-lived physiological changes. We describe the changes in the microbiota and metabolic phenotype under different timings of CR and concluded that synchronizing light-dark cycles with feeding-fasting cycles might generate a better metabolic phenotype associated with an improved gut microbiota.

## RESULTS

### Calorie-restricted mice exhibited altered metabolic outcomes and reduced inflammatory markers regardless of feeding time.

**FIG 1:**
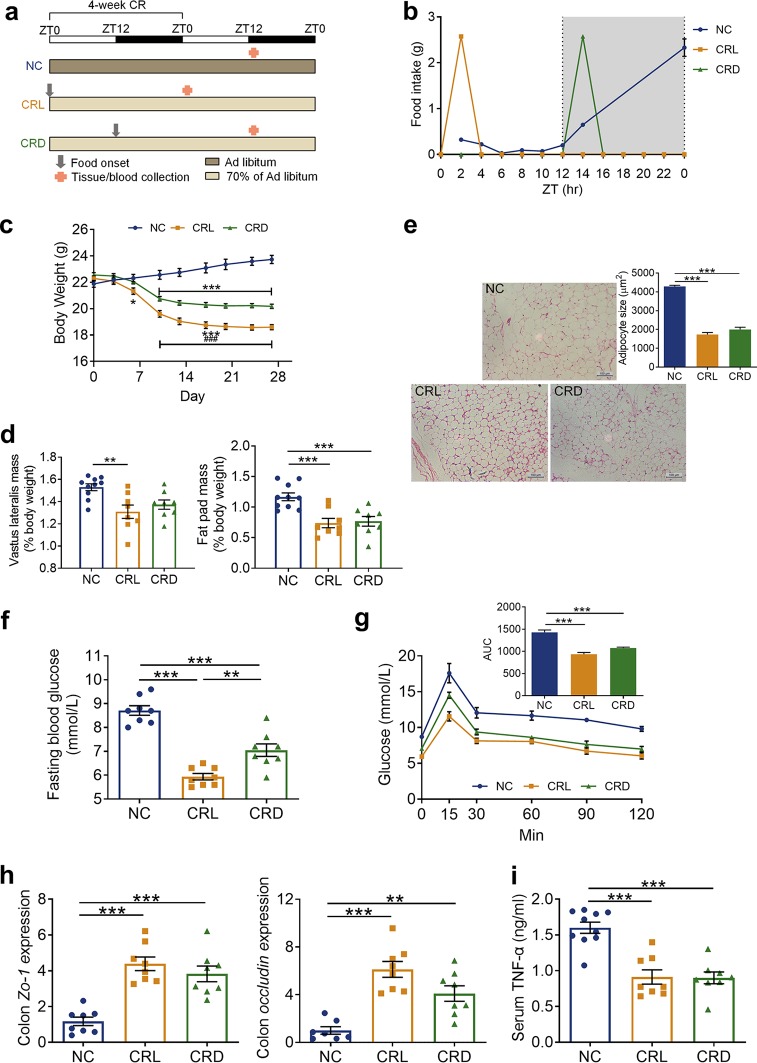
Calorie-restricted mice exhibited altered metabolic outcomes and reduced inflammatory markers regardless of feeding time. (a) Schematic of the experimental design during 4-week CR and the schedule of tissue collection in relation to the light-dark cycle and food availability after 4-week CR. The light-dark cycle is shown in the bar across the top. (b) Monitored every-2-h food intake after 4-week CR. (c) Body weight curve of each group during 4-week CR. *, *P* < 0.05; ***, *P* < 0.001 (compared to the NC mice); ###, *P* < 0.001 (compared to CRD). (d) Relative weights of vastus lateralis and fat pad by the end of 4-week CR. **, *P* < 0.01; ***, *P* < 0.001. (e) Representative H&E-stained histological sections of epipdidymal adipose tissue (eAT) from NC, CRL, and CRD mice after 4-week CR (Scale bar = 100 μm) and calculated mean cell area of adipocytes in eAT. ***, *P* < 0.001. (f) Fasting blood glucose. **, *P* < 0.01; ***, *P* < 0.001. (g) Oral glucose tolerance test (OGTT) after 4-week CR and the calculated area under the curve (AUC) of blood glucose. ***, *P* < 0.001. (h) Relative mRNA expression of Zo-1 and occludin genes in the colon after 4-week CR. **, *P* < 0.01; ***, *P* < 0.001. (i) Serum level of TNF-α after 4-week CR. ***, *P* < 0.001. NC, normal chow diet; CRL, light-phase-fed with 30% calorie restriction; CRD, dark-phase-fed with 30% calorie restriction. For NC, *n* = 10; for CRL and CRD, *n* = 8. (a and c to i) Data are presented as the mean ± SEM and analyzed using one-way ANOVA (d, e, f, h, and i) and two-way repeated-measures ANOVA (c and g), followed by a Tukey *post hoc* test.

To evaluate how a shift in feeding time impacted physiological and metabolic consequences under the CR regimen, 8-week-old male C57BL/6 mice were randomly assigned to one of the following three groups (Fig. 1a): (i) the normal chow (NC) group received *ad libitum* access to the normal chow diet, (ii) the CRL group was fed 70% of the *ad libitum* chow that was provided at the beginning of the light phase (Zeitgeber time 0 [ZT0]), and (iii) the CRD group was fed 70% of the *ad libitum* chow provided at the beginning of the dark phase (ZT12). At the end of the 4-week CR, the schedule of tissue collection in relation to the light-dark cycle and food availability was designed to ensure the same fasting duration in the CRL and CRD groups (Fig. 1a) (19).

The daily food intake of NC mice was recorded as 3.9 ± 0.52 g or 13.7 ± 1.8 kcal (mean ± standard deviation [SD]), of which 77.5% ± 5.7% was consumed during the dark/active phase (Fig. 1b). It has previously been described that calorie-restricted mice needed approximately 4 weeks to adapt to externally imposed feeding schedules and reach a relatively stable state (19); therefore, we measured the feeding behavior of our mice every 2 h after 4 weeks of CR. CRL and CRD mice consumed all their food within 2 h as soon as it was made available (Fig. 1b). This type of self-imposed feeding behavior in response to reduced calorie intake, no matter the time that the food was made available to the mice (i.e., ZT0 for CRL or ZT12 for CRD), was in agreement with results from a recently published study (19).

During the 4-week CR period, the CRL and CRD mice exhibited a decrease in body weight compared to the NC mice, but the CRL mice lost significantly more weight than did the CRD mice (Fig. 1c). By the end of the 4-week CR, the CRL mice exhibited a significant decrease in vastus lateralis mass compared to NC mice, but this was not observed in the CRD mice (Fig. 1d). The CRL and CRD mice both showed a significant decrease in fat pad weight compared to the NC mice (Fig. 1d), and hematoxylin and eosin (H&E)-stained epididymal fat of CRL and CRD mice revealed smaller adipocytes (Fig. 1e). Furthermore, the CRL and CRD mice both showed a reduction in fasting blood glucose levels compared to the NC mice (Fig. 1f) and an improvement in glucose tolerance in the oral glucose tolerance test (OGTT) compared to the NC mice (Fig. 1g). A previous study identified that hyperglycemia correlated with a loss of intestinal barrier permeability (25). To determine if intestinal permeability was also impacted by light versus dark CR, we measured the expression of two epithelial tight junction proteins zonula occludens 1 (Zo-1) and occludin at the mRNA and protein levels (26, 27). CRL and CRD mice upregulated expression of the Zo-1 and occludin genes in the colon (Fig. 1h). The integrated optical densities (IOD) of Zo-1 and occludin determined by immunohistochemistry were also significantly higher in the CRL and CRD mice than in the NC mice after the 4-week CR period (see Fig. S1 in the supplemental material), implying an improvement in intestinal barrier function in CRL and CRD mice after the 4-week CR. Moreover, the serum level of tumor necrosis factor alpha (TNF-α), which plays an essential role in gut microbiota-induced aging-associated systemic inflammation (7), was significantly reduced in the CRL and CRD mice (Fig. 1i). This reflects an attenuation of inflammation after the 4-week CR. In short, CR profoundly improved the metabolic phenotype of mice regardless of the feeding time.

10.1128/mSystems.00348-19.1 FIG S1 Immunohistochemical staining of colon and integrated optical density (IOD) analysis for Zo-1 (upper) and occludin (lower) from NC mice (left), CRL mice (middle), and CRD mice (right) after a 4-week CR. Scale bar = 50 μm. Data are presented as the mean ± standard error of the mean (SEM) and analyzed using one-way ANOVA, followed by a Tukey *post hoc* test. Download FIG S1, TIF file, 2.6 MB. Copyright © 2019 Zhang et al. 2019 Zhang et al. This content is distributed under the terms of the Creative Commons Attribution 4.0 International license.

### Dark-fed CR mice demonstrated lasting effects on metabolic phenotype and systemic inflammatory marker after switching to *ad libitum* feeding.

**FIG 2:**
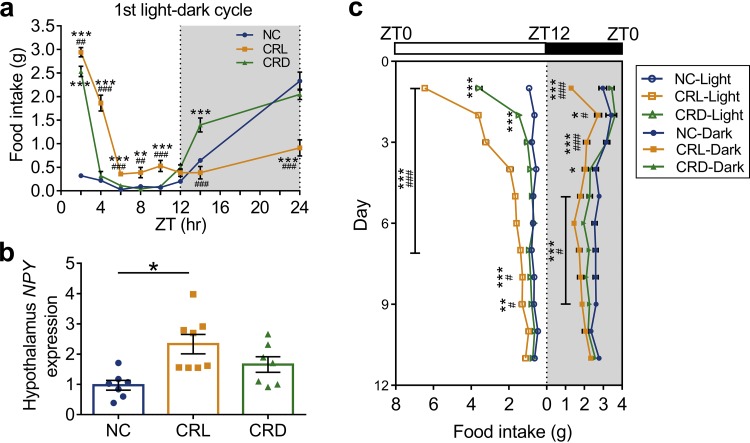
Light-fed CR mice displayed a period of food craving behavior after switching to *ad libitum* feeding. (a) Every-2-h food intake of the 1st light-dark cycle after *ad libitum* feeding. (b) Relative mRNA expression of the *NPY* gene in hypothalamus after 4-week CR. (c) Light-phase and dark-phase food intake of each group over the course of 11 consecutive light-dark cycles after *ad libitum* feeding. For the NC group, *n* = 10; for CRL and CRD, *n* = 8. Data are presented as the mean ± SEM and analyzed using one-way ANOVA (b) and two-way repeated-measures ANOVA (a and c), followed by a Tukey *post hoc* test. *, *P* < 0.05; **, *P* < 0.01; ***, *P* < 0.001 (compared to the NC mice); #, *P* < 0.05; ##, *P* < 0.01; ###, *P* < 0.001 (compared to CRD).

To investigate the lasting effects of CR with light or dark feeding, the CRL and CRD mice were switched to *ad libitum* access for 4 weeks. During the first light-dark cycle, food intake was assessed every 2 h (Fig. 2a). Both the CRL and CRD mice consumed more food than did the NC mice in the first 2 h, which might be due to a deficit of satiety during the 4-week CR. Notably, food intake in the CRL mice in the first 2 h was significantly higher than in the CRD mice (Fig. 2a). The food intake pattern of the CRD mice resembled that of the NC mice after the first 2 h, but the food intake of the CRL mice in the first light-dark cycle was significantly greater than that of the NC mice and was predominantly consumed during the light phase (Fig. 2a). Interestingly, expression of the neuropeptide Y gene (*NPY*), which is involved in appetite regulation, was significantly increased in the hypothalamus of CRL mice compared to that of the NC mice after the 4-week CR period (Fig. 2b). From the third light-dark cycle onwards, the feeding behavior of CRD mice was not significantly different from that of the NC mice, whereas it took 10 days for the CRL mice to fully recover to a normal feeding pattern (Fig. 2c and S2). Overall, these results suggest that eating during the rest period might drive a period of food craving behavior when switched to *ad libitum* feeding.

10.1128/mSystems.00348-19.2 FIG S2 Every 2-hr food intake of the 10th light-dark cycle after ad libitum. Data are presented as the mean ± SEM and analyzed using two-way repeated-measures ANOVA, followed by a Tukey *post hoc* test. Download FIG S2, TIF file, 0.2 MB. Copyright © 2019 Zhang et al. 2019 Zhang et al. This content is distributed under the terms of the Creative Commons Attribution 4.0 International license.

**FIG 3:**
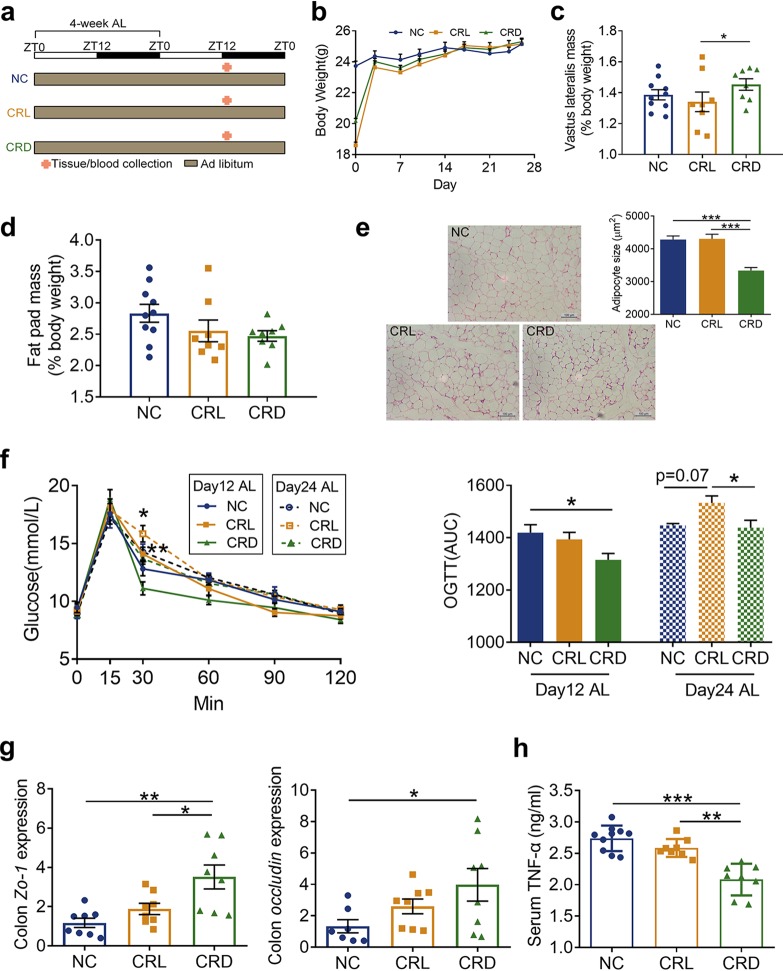
Dark-fed CR mice demonstrated lasting effects on the metabolic phenotype and systemic inflammatory marker after switching to *ad libitum* feeding. (a) Schematic of the experimental design during 4-week *ad libitum* (AL) feeding and the schedule of final tissue collection in relation to the light-dark cycle and food availability after 4-week AL feeding. The light-dark cycle is shown in the bar across the top. (b) Body weight curve of each group during 4-week *ad libitum* feeding. (c) Relative weight of vastus lateralis by the end of 4-week *ad libitum* feeding. (d) Relative weight of fat pad by the end of 4-week *ad libitum* feeding. (e) Representative H&E-stained histological sections of eAT from NC, CRL, and CRD mice after 4-week *ad libitum* feeding (scale bar = 100 μm), and the calculated mean cell area of adipocytes in eAT. (f) OGTTs performed at 12 and 24 days after the *ad libitum* feeding (day 12 AL and day 24 AL) and the calculated area under the curve (AUC) of blood glucose. *, *P* < 0.05, CRL-day 24 compared to CRD-day 24; **, *P* < 0.01, CRL-day 12 compared to CRD-day 12. (g) Relative mRNA expression of Zo-1 and occludin genes in the colon after 4-week *ad libitum* feeding. (h) Serum level of TNF-α after 4-week *ad libitum* feeding. For the NC mice group, *n* = 10; for CRL and CRD mice, *n* = 8. Data are presented as the mean ± SEM and analyzed using one-way ANOVA (c to e, g, and h) and two-way repeated-measures ANOVA (b and f), followed by a Tukey *post hoc* test. *, *P* < 0.05; **, *P* < 0.01; ***, *P* < 0.001.

By the end of the 4-week *ad libitum* period, the body weights of CRL and CRD mice rebounded to a level identical to that of the NC mice (Fig. 3a and b). The vastus lateralis mass of CRL exhibited no significant difference with that of the NC mice, but it was significantly lower than that in the CRD mice (Fig. 3c). Although the fat pad weight of the CRD mice only showed a decrease in trend compared to the NC and CRL mice (Fig. 3d), H&E-stained epididymal fat revealed that the CRD mice still had smaller adipocytes (Fig. 3e). OGTTs were performed at 12 and 24 days after the switch to *ad libitum* feeding. Interestingly, the improvement in glucose tolerance (measured by the OGTT) in the CRD mice during the CR phase was maintained until day 12 compared to the NC mice. However, in the CRL mice, there was no improvement in glucose tolerance at day 12 compared to the NC mice, and on day 24, the CRL mice showed a deterioration in OGTT response (Fig. 3f). In addition, the CRD mice continued to maintain the significant increase in Zo-1 and occludin expression at both mRNA and protein levels in the colon (Fig. 3g and S3), as well as the lower serum TNF-α level compared to that in the NC and CRL mice even after 4 weeks of *ad libitum* feeding (Fig. 3h). These observations suggest that the beneficial effects of improved intestinal barrier function and alleviation of the systemic inflammatory marker induced by CR persisted in the CRD mice after the 4-week *ad libitum* period.

10.1128/mSystems.00348-19.3 FIG S3 Immunohistochemical staining of colon and integrated optical density (IOD) analysis for Zo-1 (upper) and occludin (lower) from NC mice (left), CRL mice (middle), and CRD mice (right) after a 4-week ad libitum period. Scale bar = 50 μm. Data are presented as the mean ± SEM and analyzed using one-way ANOVA, followed by a Tukey *post hoc* test. Download FIG S3, TIF file, 2.6 MB. Copyright © 2019 Zhang et al. 2019 Zhang et al. This content is distributed under the terms of the Creative Commons Attribution 4.0 International license.

Taken together, these data indicate that protective effects on fat accumulation, glucose metabolism, and intestinal barrier function and on attenuation of the systemic inflammatory marker were maintained in the CRD mice over prolonged times after shifting from CR to *ad libitum* access. On the other hand, disrupted feeding behavior in the CRL mice was observed after switching to *ad libitum* access, and the physiological changes of CR were short-lived in the CRL mice.

### Light-fed CR mice showed different fecal microbiota structural changes from those of dark-fed mice.

Microbial oscillations are mainly driven by the food consumption rhythmicity (12–14); therefore, we collected fecal samples on the day before treatment and at two time points (ZT0 and ZT12) of the day after the 4-week CR and of days 2, 11, 19, and 26 after switching to *ad libitum* feeding and profiled the gut microbiota by sequencing the V3-V4 regions in the 16S rRNA gene. Since CRL mice consumed all their food in the light phase during the 4-week CR period (Fig. 1b), we compared the gut microbiota of the CRL mice at ZT0 with that of the NC and CRD mice at ZT12, or that of the CRL mice at ZT12 with that of the NC and CRD mice at ZT0 after 4-week CR, in order to keep the same time before or after the onset of meal time. The same plan was followed for analyzing the gut microbiota for day 2 AL (day 2 after switching to *ad libitum* feeding) considering that most of the food in CRL mice was still being consumed during the light phase in the first light-dark cycle after switching to *ad libitum* feeding (Fig. 2a and c). For days 11, 19, and 26 AL, we compared the gut microbiota between the groups at the same time points, as the CRL mice had changed their feeding pattern to one similar to that of the NC mice after 10 days of *ad libitum* access.

**FIG 4:**
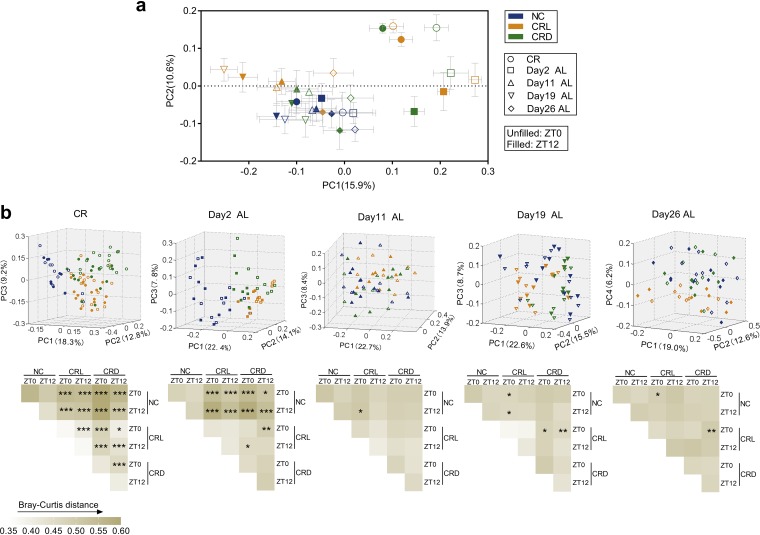
Light-fed CR mice showed different fecal microbiota structural changes compared to dark-fed mice. (a) Principal-coordinate analysis (PCoA) based on Bray-Curtis distance of all the samples collected at two time points (ZT0 and ZT12) of the day after the 4-week calorie restriction (CR) period and of days 2, 11, 19, and 26 after *ad libitum* feeding. (b) Top, PCoA based on Bray-Curtis distance. Bottom, heatmap of Bray-Curtis distances between each group and permutational multivariate analysis of variance (PERMANOVA; 9,999 permutations) were shown separately on the day after 4-week CR and days 2, 11, 19, and 26 AL (from left to right). *, *P* < 0.05; **, *P* < 0.01; ***, *P* < 0.001.

Before the treatment, no significant difference in gut microbiota structure was observed among the three groups (Fig. S4). Four-week CR had no significant effect on the diversity and richness of the gut microbiota among the three groups (Fig. S5a). Principal-coordinate analysis (PCoA) and permutational multivariate analysis of variance (PERMANOVA) based on the Bray-Curtis distance show that after 4 weeks of CR, the gut microbiota structure of CRL and CRD significantly diverged from that of the NC mice mainly along the axis representing the first principal component (PC1), irrespective of sample collection time (ZT0 or ZT12), reflecting that the amount of food intake was the predominant factor in shifting the gut microbiota (Fig. 4a and b). However, the shift in feeding time also exerted a significant change in gut microbiota structure, as shown by the distancing in clustering of gut microbiota isolated from CRL mice compared to CRD mice (Fig. 4b). On the other hand, the variation in the gut microbiota at ZT0 and ZT12 was more pronounced in both the CRL and CRD mice than in the NC mice (Fig. 4b), which may be due to the self-imposed 2-h daily restriction of food intake and the extended fasting period between meals. Interestingly, it was noted that CRL-ZT0 and CRD-ZT12 were most similar in gut microbiota structure, as shown by the PCoA plot and the comparison on distance (Fig. 4a and S6).

10.1128/mSystems.00348-19.4 FIG S4 16S rRNA profiles of baseline samples. Top, principal-coordinate analysis (PCoA) based on Bray-Curtis distance. Bottom, heatmap of Bray-Curtis distances between each group and permutational multivariate analysis of variance (PERMANOVA; 9,999 permutations). Download FIG S4, TIF file, 0.8 MB. Copyright © 2019 Zhang et al. 2019 Zhang et al. This content is distributed under the terms of the Creative Commons Attribution 4.0 International license.

10.1128/mSystems.00348-19.5 FIG S5 Alpha diversity of the gut microbiota. (a to e) The observed OTUs (left), Shannon diversity index (middle), and Faith’s phylogenetic diversity values (right) after 4-week calorie restriction (CR) (a) and on days 2 (b), 11 (c), 19 (d), and 26 (e) after *ad libitum* (AL). The sampling level is 12,300. Data are presented as the mean ± SEM and analyzed using two-way repeated-measures ANOVA, followed by a Tukey *post hoc* test. **, *P* < 0.01. Download FIG S5, TIF file, 1.3 MB. Copyright © 2019 Zhang et al. 2019 Zhang et al. This content is distributed under the terms of the Creative Commons Attribution 4.0 International license.

10.1128/mSystems.00348-19.6 FIG S6 Bray-Curtis distance between CRL and CRD mice compared at two time points after a 4-week CR period. Data are presented as the mean ± SEM and analyzed using one-way ANOVA, followed by a Tukey *post hoc* test. ***, *P* < 0.001. Download FIG S6, TIF file, 0.2 MB. Copyright © 2019 Zhang et al. 2019 Zhang et al. This content is distributed under the terms of the Creative Commons Attribution 4.0 International license.

At day 2 AL, alpha diversity was significantly reduced in CRL-ZT0 mice compared to that in the NC-ZT12 and CRD-ZT12 mice (Fig. S5b), which might be associated with the increased food intake in CRL mice after switching to *ad libitum* feeding (Fig. 2a). From the score plot of the PCoA, the gut microbiota of the CRL and CRD mice was still significantly different from that of the NC mice, regardless of the collection time (ZT0 or ZT12) (Fig. 4a and b). We also noticed significant differences between the CRL-ZT0 and CRD-ZT12 mice, as well as between the CRL-ZT12 and CRD-ZT0 mice (Fig. 4b). Interestingly, the significant variation at ZT0 and ZT12 in the gut microbiota in both CRL and CRD mice disappeared from day 2 onwards (Fig. 4a and b). Furthermore, the gut microbiota of the CRD mice moved close to that of the NC mice in the PCoA plot and showed no significant difference from that of the NC mice from day 11 onwards (Fig. 4a and b). However, the gut microbiota of the CRL mice at days 11 and 19 AL moved to the left part of the PC1 axis, which changed in a direction opposite from that of the CR stage (Fig. 4a). Significant differences in the gut microbiota were still observed between the CRL and NC mice and/or the CRD mice from day 11 onwards, and from the PCoA plot, the gut microbiota of CRL mice still separated from that of the NC and CRD mice along PC4 at day 26 AL (Fig. 4b).

### Shifting feeding time impacts fecal microbiota composition.

**FIG 5:**
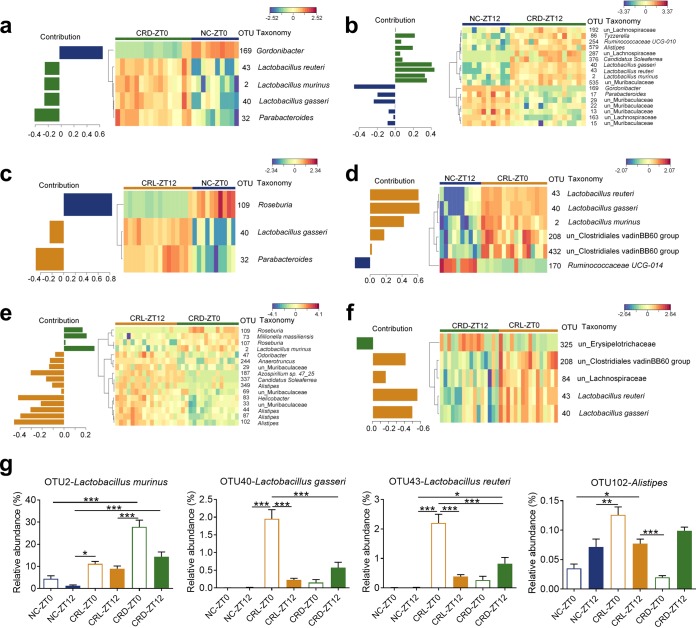
Shifting feeding time impacts fecal microbiota composition. (a to f) Contribution plots and heatmaps of key OTUs for separating the gut microbiota among different groups after 4-week CR are shown in NC-ZT0 and CRD-ZT0 (a), NC-ZT12 and CRD-ZT12 (b), NC-ZT0 and CRL-ZT12 (c), NC-ZT12 and CRL-ZT0 (d), CRL-ZT12 and CRD-ZT0 (e), and CRL-ZT0 and CRD-ZT12 (f). The color of the spots represented the relative abundance (normalized and log transformed) of the OTUs in each group. OTUs were arranged according to clusters based on the complete linkage method. The taxonomy of each OTU is shown on the right. See Fig. S7 for sPLS-DA models. (g) Relative abundances of three *Lactobacillus* OTUs and one *Alistipes* OTU after 4-week CR. For NC group, *n* = 10; for CRL and CRD groups, *n* = 8. Data are presented as the mean ± SEM and analyzed using two-way repeated-measures ANOVA, followed by a Tukey *post hoc* test. ***, *P* < 0.001; **, *P* < 0.01; *, *P* < 0.05.

The sparse partial least squares discriminant analysis (sPLS-DA) (28) was utilized to identify members of the gut microbiota that were affected by CR and/or the shift in feeding time after the 4-week CR. First, the NC and CRD mice were compared to investigate the effects of CR on gut microbiota composition (Fig. 5a and b and S7a and b). Four OTUs changed in a similar way at ZT0 and ZT12, including three *Lactobacillus* OTUs (OTU2, OTU40, and OTU43) which were significantly enriched in the CRD mice, while Gordonibacter (OTU169) was depleted in CRD mice (Fig. 5a and b). Parabacteroides (OTU32) was enriched in the CRD-ZT0 mice compared to the NC-ZT0 mice (Fig. 5a), but OTU17, belonging to the same genus, was reduced in CRD-ZT12 mice compared to NC-ZT12 mice (Fig. 5b). These results substantiate our previous findings that bacteria belonging to *Lactobacillus* become prevalent phylotypes in CR mice (8, 9). Second, the NC and CRL mice were compared to identify OTUs affected by CR with reversal of feeding time (Fig. 5c and d and S7c and d). Lactobacillus gasseri (OTU40) was enriched in CRL mice irrespective of sample collection time (ZT0 or ZT12), whereas two other *Lactobacillus* OTUs (OTU2 and OTU43) were significantly increased in CRL-ZT0 mice compared to NC-ZT12 mice (Fig. 5c and d). This enrichment of *Lactobacillus* spp. was observed in both the CRL and CRD mice. It was noted that some OTUs only responded to either CRL or CRD in comparison to the NC mice, such as Roseburia spp. (OTU109), which were decreased in CRL-ZT12 mice compared to NC-ZT0 mice (Fig. 5c) but were not found in the CRD mice compared to the NC mice. This indicated that gut microbiota is not only affected by how much is eaten but also by when the food is consumed. Third, we compared the CRL and CRD mice, which were both fed 70% of the *ad libitum* intake to determine the effects of shifting feeding time on gut microbiota composition (Fig. 5e and f and S7e and f). The relative abundance of *L. murinus* (OTU2) was higher in the CRD-ZT0 than in the CRL-ZT12 mice (Fig. 5e), while the relative abundances of L. gasseri (OTU40) and Lactobacillus reuteri (OTU 43) were lower in CRD-ZT12 mice than in CRL-ZT0 mice (Fig. 5f). Notably, the relative abundance of OTU2 was elevated to an average of 28% ± 3% in CRD-ZT0 mice (Fig. 5g). Also, the relative abundances of two *Roseburia* OTUs (OTU107 and OTU109) were higher in the CRD-ZT0 mice than in the CRL-ZT12 mice (Fig. 5e), while the relative abundances of Alistipes (OTU44, OTU87, OTU102, and OTU349), Odoribacter (OTU47), and Helicobacter (OTU83) spp. were higher in the CRL-ZT12 mice than in the CRD-ZT0 mice (Fig. 5e).

10.1128/mSystems.00348-19.7 FIG S7 The optimal classification performance of the sPLS-DA model related to FIG 5. (a to f) The plots of the classification error rate of different components averaged across the leave-one-out cross-validation for maximum distances (left side of each panel), and the sPLS-DA plots based on OTU table (right side of each panel) for separating the gut microbiota of NC-ZT0 and CRD-ZT0 (a), NC-ZT12 and CRD-ZT12 (b), NC-ZT0 and CRL-ZT12 (c), NC-ZT12 and CRL-ZT0 (d), CRL-ZT12 and CRD-ZT0 (e), and CRL-ZT0 and CRD-ZT12 (f). Download FIG S7, TIF file, 0.6 MB. Copyright © 2019 Zhang et al. 2019 Zhang et al. This content is distributed under the terms of the Creative Commons Attribution 4.0 International license.

**FIG 6:**
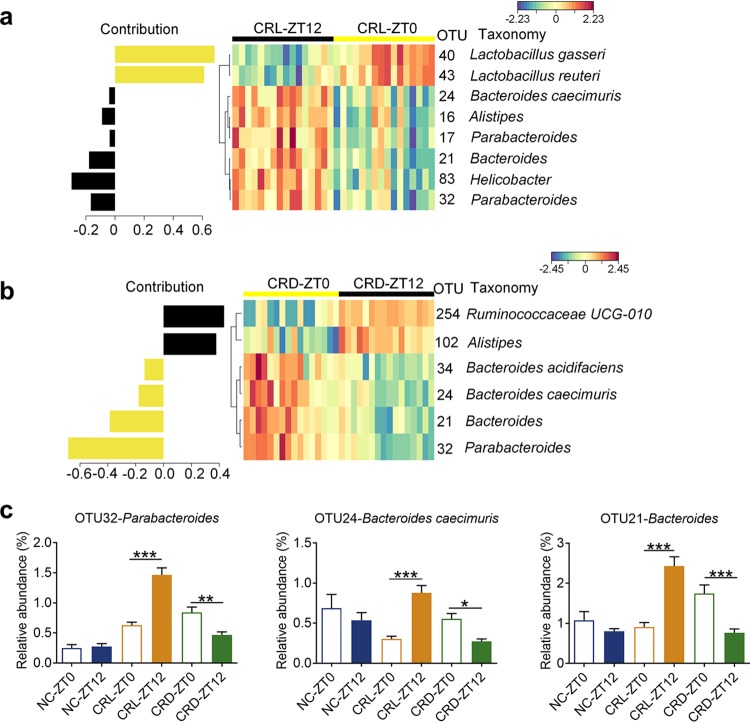
The variation of gut microbiota at ZT0 and ZT12 in two types of CR mice. (a and b) The contribution plot of OTUs and heat maps of key OTUs for separating the gut microbiota of CRL-ZT0 and CRL-ZT12 (a) and CRD-ZT0 and CRD-ZT12 (b). See Fig. S8 for sPLS-DA models. (c) Relative abundances of 3 OTUs (OTU32, OTU24, and OTU21). For the NC group, *n* = 10; for the CRL and CRD groups, *n* = 16. Data are presented as the mean ± SEM and analyzed using two-way repeated-measures ANOVA, followed by a Tukey *post hoc* test. *, *P* < 0.05; **, *P* < 0.01; ***, *P* < 0.001.

Additionally, we compared the variation of ZT0 and ZT12, respectively, in CRL and CRD mice to identify OTUs that were affected by the self-imposed feeding-fasting cycles (Fig. 6 and S8). OTU40 (L. gasseri) and OTU43 (L. reuteri) were significantly higher in the CRL-ZT0 mice than in the CRL-ZT12 mice, and a distinct trend could be observed in CRD mice (Fig. 6a and 5g), which might suggest that they were at higher levels after longer fasting duration. The abundances of OTU32 (*Parabacteroides*), OTU24 (Bacteroides caecimuris), and OTU21 (*Bacteroides*) were significantly higher in CRD-ZT0 mice than in CRD-ZT12 mice, in line with the trends seen in the NC mice, while the CRL mice showed adverse variation at ZT0 and ZT12 (Fig. 6). This might suggest that they were at lower levels after longer fasting durations. The CRL-ZT0 and CRD-ZT12 mice were most similar in terms of both the compositional analysis and the comparison on distance (Fig. S6), suggesting that it is the fasting state that is driving the differences in gut microbiota.

10.1128/mSystems.00348-19.8 FIG S8 The optimal classification performance of the sPLS-DA model related to FIG 6. (a and b) The plots of the classification error rate of different components averaged across the leave-one-out cross-validation for maximum distances (left), and the sPLS-DA plots based on OTU table (right) for separating the gut microbiota of CRL-ZT0 and CRL-ZT12 (a) and CRD-ZT0 and CRD-ZT12 (b). Download FIG S8, TIF file, 0.1 MB. Copyright © 2019 Zhang et al. 2019 Zhang et al. This content is distributed under the terms of the Creative Commons Attribution 4.0 International license.

Furthermore, as mentioned earlier, the gut microbiota structures of the CRL and NC mice were significantly different during the 4-week *ad libitum* feeding, and particularly at day 19 AL (Fig. 4a). Next, we also used sPLS-DA model to identify the phylotypes that showed differences between CRL and the NC mice at day 19 AL (Fig. S9). The compositional difference between the CRL and NC mice was that the relative abundances of *Alistipes* spp. (OTU102) and L. gasseri (OTU40) were higher in CRL mice than in the NC mice (Fig. S9). The enrichment of *Alistipes* spp. (OTU102) was also observed in CRL after 4-week CR and at day 2 AL (Fig. 5g and S10a). Interestingly, three *Lactobacillus* OTUs (OTU2, OTU40, and OTU43) showed different responses in CRL and CRD mice, not only after the 4-week CR, but also during the subsequent *ad libitum* period. The relative abundance of *L. murinus* (OTU2) was rapidly reduced in CRD at day 2 AL and did not differ among the three groups from day 11 onwards (Fig. 5g and S10), suggesting a strong correlation with CR. However, L. gasseri (OTU40) and L. reuteri (OTU 43) were elevated in the CRD mice at day 2 AL compared to the preceding CR period and were higher in both the CRL and CRD mice than in the NC mice at day 2 AL (Fig. 5g and S10). These OTUs remained significantly higher in the CRL mice than in the NC mice at day 11 AL and day 19 AL, but this was not observed in the CRD mice (Fig. S10).

10.1128/mSystems.00348-19.9 FIG S9 The difference of gut microbiota between CRL and NC at ZT0 on day 19 after *ad libitum*. The plot of the classification error rate of different components averaged across the leave-one-out cross validation for maximum distances and sPLS-DA plot based on OTU table (top) and contribution plot of OTUs and heat map of key OTUs (bottom) for separating the gut microbiota of CRL-ZT0 and NC-ZT0. Download FIG S9, TIF file, 0.2 MB. Copyright © 2019 Zhang et al. 2019 Zhang et al. This content is distributed under the terms of the Creative Commons Attribution 4.0 International license.

10.1128/mSystems.00348-19.10 FIG S10 Relative abundance of three *Lactobacillus* OTUs and one *Alistipes* OTU. (a to d) OTU2 (left), OTU40 (middle-left), OTU43 (middle-right), and OTU102 (right) in days 2 (a), 11 (b), 19 (c), and 26 (d) after *ad libitum*. For the NC group, *n* = 10; for CRL and CRD, *n* = 8. Data are presented as the mean ± SEM and analyzed using two-way repeated-measures ANOVA, followed by a Tukey *post hoc* test. ***, *P* < 0.001; **, *P* < 0.01; *, *P* < 0.05. Download FIG S10, TIF file, 0.9 MB. Copyright © 2019 Zhang et al. 2019 Zhang et al. This content is distributed under the terms of the Creative Commons Attribution 4.0 International license.

Collectively, 16S rRNA amplicon sequencing analysis from CRL and CRD mouse gut microbiota revealed that CR alone elicited a substantial influence on microbiota composition; however, the time of feeding also contributed.

## DISCUSSION

Our study found that the shift in feeding time impacts CR-induced effects, including physiological changes, modulation of gut microbiota, and anti-inflammation changes, all of which may contribute to improved health and extended longevity (8, 9, 19, 20). Two major characteristics of the CR regimen are (i) the reduction in total calorie intake without malnutrition, and (ii) periodicity in availability of food (e.g., self-imposed 2-h daily restriction of food intake in mice). Previous studies paid much attention to the former but ignored the latter. Feeding parameters (amount, duration, and timing of food access) strongly and differently impact the beneficial health outcomes (19), but the timing of food access in many CR studies is not even stated or specified (3, 29, 30). The current work clarified that when the food is made available (dark or light phase) was essential and influenced the outcomes of CR.

Previous studies showed that limiting the daily food intake of an isocaloric diet to a smaller time window of 8 to 12 h per day was sufficient to improve health and longevity in mice, and restricting both feeding duration and caloric intake (i.e., CR) exhibited much more beneficial effects (1, 11, 20). In the current work, light-fed mice lost significantly more weight than did their dark-fed companions and exhibited muscle loss compared to the controls after the 4-week CR. This difference in body weight and muscle mass under the two CR conditions reinforces the idea that feeding time is critical, since these mice consumed the same amount of food each day. Previous work showed that the clock in skeletal muscle appeared to be more sensitive to feeding patterns, resulting in large changes in gene and protein expression (31). Future work on the timing of food access and changes in muscle might provide insights into its molecular mechanisms of action. During the subsequent *ad libitum* feeding period, mice that had previously been light fed demonstrated disrupted feeding behavior, short-lived physiological changes, and even a deterioration of glucose tolerance. This suggests that in addition to the amount of food and feeding duration, when to eat (light phase or dark phase) also had an important influence on metabolic health. In future studies, it would be interesting to investigate whether the timing of CR can have effects on life span and aging through lifelong CR experiments.

CR profoundly improves metabolic phenotype through its potential anti-inflammatory effects (32, 33). In agreement with this, a significant reduction in inflammatory markers and improvement in intestinal barrier function were observed in our CR mice. A recent study on the anti-inflammatory effects of CR showed that CR-induced microbiota alterations dictated the tone of the immune response and contributed to the improvements by the reduction of endotoxin in mice (5), but which member of the gut microbiota acted as a determinant of the beneficial effects was not identified. Here, we observed that the timing of CR impacts the structure and composition of fecal microbiota in mice, and a higher abundance of *L. murinus* was noted in dark-fed CR mice than in their light-fed companions. Previously, we isolated a strain designated *L. murinus* CR147, which could represent the most abundant *Lactobacillus* OTU enriched by CR. The similarity of the representative sequence of OTU2 in the current work and the V3-V4 regions of the 16S rRNA gene sequence of *L. murinus* CR147 was 100%. Since this strain has been shown to protect the gut barrier and alleviate aging-associated inflammation (9), promoting this OTU might be informative for the better metabolic consequences after switching to *ad libitum* feeding. Furthermore, members of the dominant genus *Lactobacillus* might take advantage of the resource and make the gut environment unfavorable to pathogenic or detrimental bacteria (34, 35). Moreover, other bacteria might also contribute to the beneficial effects of dark-fed CR. *Roseburia* spp. were also enriched in dark-fed CR mice, and a member of this genus was reported to produce butyrate, decrease levels of inflammatory markers, and improve gut barrier function (36). Members of the gut microbiota that increased in light-fed mice, such as *Alistipes*, *Odoribacter*, and *Helicobacter* spp., were reported to be correlated with some diseases. Members of the genus *Alistipes* were associated with the pain in patients with irritable bowel syndrome (37). Higher abundances of *Alistipes* and *Odoribacter* spp. were found in grid floor stress-induced BALB/c mice and mice fed a high-fat diet (38, 39). Additionally, *Helicobacter* infection is known to cause gastric cancer (40). Light-fed CR may promote detrimental shifts in the microbiota that could elicit negative consequences on metabolic outcomes.

The genus *Lactobacillus*, which is widely used as probiotics, has been shown in many studies to protect against pathogen-induced gut barrier disruption and to alleviate inflammation (9, 41–45). However, two recent clinical studies reported that Lactobacillus rhamnosus GG did not bring better outcomes (46, 47), and L. reuteri was even reported to be obesogenic in humans (48). In the current work, three *Lactobacillus* OTUs were enriched by CR but showed different responses in light-fed and dark-fed mice, not only after the 4-week CR, but also during the follow-up *ad libitum* period. Specifically, L. gasseri (OTU40) and L. reuteri (OTU43) still presented a higher abundance in CRL mice after *ad libitum* feeding, concomitant with the short-lived physiological changes. The similarities of the representative sequences of OTU40 and OTU43 with the V3-V4 regions of the 16S rRNA gene sequence of *L. murinus* CR147 were 88% and 92%, respectively. In this regard, future work on probiotics is required to explore the strain-specific protective mechanisms for the development of targeted therapies (45, 49, 50).

Comparing the two time points of the fecal microbiota in the two CR groups provides an opportunity to find that some OTUs, such as *Lactobacillus*, *Parabacteroides*, and *Bacteroides*, featured distinct phases between dark- and light-fed groups, all of which were highly robust oscillations reported previously (12, 18). Prolonged fasting duration might drive the differences in gut microbiota, as it influences the nutrient availability to bacteria (16). Our microbial analysis was based on the stool 16S rRNA gene sequencing as a proxy of gastrointestinal composition, which may yield limited conclusions (51). For future studies, it would be of utmost significance to profile the microbiota along the entirety of the intestinal tract to accomplish a comprehensive assessment of CR effects on mucosal and luminal microbiota and the mechanism of blooming of some bacteria.

Many people who diet to lose weight find it logistically difficult to implement CR over their lifetime and thereby undergo excessive weight regain cycles. Research previously investigated the “yo-yo effect,” based on the nutrition quality changing from a high-fat diet to normal chow diet, and demonstrated that the microbiome plays a key role in this phenomenon (52). The CR regimen focuses on the quantity of diet, and the current work shows that when to eat might act as a modulator of gut microbiota, impacting the lasting effects of CR. The causal role of CR-induced microbiota modulation and metabolic health has been established through microbiota transplantations (5). In addition, the role of the time-shifted microbiota in the metabolic imbalances observed in jet-lagged mice was corroborated upon fecal transplantation (12). Further work is needed to build upon the existing findings to identify the causative linkage between timed CR-induced gut microbes and metabolic outcomes.

Timing of food access should be taken into careful consideration in a CR regimen. Erratic daily eating patterns are prevalent in modern society, with people eating more frequently throughout the day and with a bias toward late-night eating (53). In light of the fact that humans naturally spend the light phase in the active/feeding state and the dark phase in the resting/fasting state, avoiding eating close to or during the rest period when people are on a diet might generate better metabolic outcomes. Our work strengthens the evidence for using “when to eat” as an intervention to improve health during calorie restriction.

## MATERIALS AND METHODS

### Ethical approval.

All animal experimental procedures were approved by the Institutional Animal Care and Use Committee (IACUC) of the School of Life Sciences and Biotechnology of Shanghai Jiao Tong University (no. 2014005).

### Animals, housing, and experimental design.

Specific-pathogen-free (SPF) 4-week-old male C57BL/6J mice were purchased from SLAC, Inc. (Shanghai, China). The mice arrived at the animal center of Shanghai Jiao Tong University at the same time and were single housed and acclimatized to the animal facility environment for 4 weeks before experimentation; the mice were therefore 8 weeks old at the beginning of the experiments. Mice were randomly assigned to one of the following three groups and individually caged for an 8-week trial: (i) normal chow diet (NC, *n* = 10), with 8-week *ad libitum* access to food; (ii) normal chow diet with 30% calorie restriction, with food provided at the beginning of the light phase (CRL, *n* = 16) and 4-week calorie restriction followed by 4-week *ad libitum* access; (iii) normal chow diet with 30% calorie restriction, with food provided at the beginning of the dark phase (CRD, *n* = 16) and 4-week calorie restriction followed by 4-week *ad libitum* access (Fig. 1a and 3a). For the NC group, food was available in excess of normal consumption at all time; for the two CR groups, 70% of the *ad libitum* intake was provided once daily at either the beginning of the night or the day. The normal chow diet (22.1% protein, 5.3% fat, 3% fiber, 3.52 total kcal/g; product number P1101F) was purchased from SLAC, Inc. The daily food consumption of the NC group was recorded over 1 week and averaged to determine the amount of food for the two CR groups in the following week. All mice were single housed during the study to avoid any competition effects, ensuring that each mouse in the two CR groups had access to 70% of the intake of the NC group (3, 9). All mice were kept under a strict 24-h light-dark cycle, with lights being turned on from 7 a.m. to 7 p.m. (Zeitgeber time 0 [ZT0], 7 a.m., lights on; ZT12, 7 p.m., lights off).

### Sample collection.

During the 4-week CR, body weight was measured twice weekly. The Zeitgeber time of when the body weight was measured, when the OGTT was performed, when blood samples were taken, and when the mice were dissected was before the onset of food availability for each group (Fig. 1a), as previously described (19). At the end of the 4-week CR, half of the animals were sacrificed, and adipose tissues (epididymal, mesenteric, subcutaneous inguinal, and retroperitoneal), vastus lateralis muscles, colon, and hypothalamus, etc., were collected and weighed. Blood samples were collected from the orbital vascular plexus, and the serum was isolated for TNF-α assays. After a 4-week *ad libitum* feeding period, as the two CR groups had recovered to normal feeding patterns after 10 days of *ad libitum* feeding, the rest of the animals were sacrificed at the same Zeitgeber time after fasting for 6 h, and tissue and blood samples were collected (Fig. 3a). The animals did not receive food on the day of dissection. All samples were stored at –80°C until analysis.

### Histopathology.

Fresh epididymal fat pads were fixed with 4% paraformaldehyde and then embedded in paraffin. Subsequently, 4-μm sections were stained by hematoxylin and eosin (H&E). Digital images of sections were acquired with a Leica DMRBE microscope. Adipocyte size (cross-sectional area) was counted using Image Pro Plus 6.0. For each mouse, areas of adipocytes were determined in at least three histological sections and 300 total adipocytes.

### Oral glucose tolerance test.

The Zeitgeber time of when the OGTT was performed after the 4-week CR was before the onset of food availability for each group, while that after 4-week *ad libitum* feeding was after 6 h fasting for all groups (54). Glucose was administered to mice by oral gavage at a dose of 2.0 g/kg of body weight. Blood glucose concentrations, in samples collected from the tip of the tail vein, were determined at 0, 15, 30, 60, 90, and 120 min after glucose challenge (Accu-Chek 381 Performa; Roche, USA).

### RNA isolation and RT-qPCR.

Total RNA was extracted from the colon and hypothalamus using an RNeasy lipid tissue minikit (catalog no. 74804; Qiagen, Germany), according to the manufacturer’s protocol. Subsequent to the verification of RNA quality (gel electrophoresis), 1 μg of each total RNA sample (determined spectrophotometrically) was treated with RNase-free DNase I (catalog no. 18068-015; Invitrogen, USA). First-strand cDNA was synthesized using random hexanucleotide primers and SuperScript III reverse transcriptase (catalog no. 18080-051; Invitrogen, USA). Forward and reverse primers were added to SYBR green I PCR supermix (Bio-Rad) to amplify the Zo-1, occludin, and *NPY* genes. Real-time quantitative PCR (RT-qPCR) was performed on a LightCycler 96 system (Roche Applied Science). The PCR conditions were 95°C for 3 min, followed by 40 cycles of 95°C for 20 s, 56°C for 30 s, and 72°C for 30 s, and plate reads for 5 s. Gene expression levels were determined using the comparative ΔΔ*C_T_* method (2^−ΔΔ^*^CT^* method), with the β-actin gene serving as the reference gene. Forward (F) and reverse (R) primer sequences are as follows: Zo-1 gene, F-ACCCGAAACTGATGCTGTGGATAG, and R-AAATGGCCGGGCAGAACTTGTGTA; occludin gene, F-ATGTCCGGCCGATGCTCTC and R-TTTGGCTGCTCTTGGGTCTGTAT; *NPY*, F-ATGCTAGGTAACAAGCGAATGG and R-TGTCGCAGAGCGGAGTAGTAT; and β-actin gene, F-GGCTGTATTCCCCTCCATCG and R-CCAGTTGGTAACAATGCCATGT.

### Immunohistochemistry.

After deparaffinization and processing for antigen retrieval, the blank colon sections were incubated overnight at room temperature with anti-rabbit Zo-1 antibody (1:500, catalog no. GB11195; Servicebio, China) and occludin antibody (1:200, catalog no. GB11149; Servicebio). Sections were then incubated with anti-rabbit IgG secondary antibody (KPL, MA, USA) for 50 min at room temperature. After washing with phosphate-buffered saline (PBS), the DAB Envision kit (catalog no. K5007; Dako, Copenhagen, Denmark) was used to develop color; Zo-1 and occludin appear brown, while nuclei are blue. Images of each colon section were obtained in tripartitely by experienced staff who were blind to the experiment under ×400 magnification using a Leica DMRBE microscope and were analyzed using Image Pro Plus 6.0, according to the method previously described (55). The integrated optical density (IOD) values were log_10_ transformed.

### Serum TNF-α measurements.

Blood samples were centrifuged at 4,000 rpm and 4°C for 15 min, and the serum was stored at –80°C until further analyses. Enzyme-linked immunosorbent assays (ELISAs) for tumor necrosis factor-alpha (TNF-α) were purchased from R&D Systems, Inc. (MHSTA50; Minneapolis, MN, USA), and were conducted according to the manufacturer’s instructions.

### Fecal DNA extraction and 16S rRNA gene V3-V4 region sequencing.

Microbial DNA was extracted, as previously described (56), from fecal samples collected the day before treatment (for baseline samples, NC, *n* = 10; CRL, *n* = 16; and CRD, *n* = 16) and at two time points (ZT0 and ZT12) of the day after 4-week CR (NC, *n* = 10; CRL, *n* = 16; and CRD, *n* = 16) and of days 2, 11, 19, and 26 after switching to *ad libitum* feeding (NC, *n* = 10; CRL, *n* = 8; and CRD, *n* = 8). A total of 334 samples were sequenced on the MiSeq system (Illumina, Inc., USA) with the MiSeq reagent kit v3 (600 cycles, catalog no. MS-102-3033; Illumina). A sequencing library of the V3-V4 regions of the 16S rRNA gene was constructed according to the manufacturer’s instructions (Part # 15044223 Rev. B; Illumina Inc., USA) with some modifications as previously published (57).

### Analysis of 16S rRNA V3-V4 sequencing data.

High-quality sequence alignments, sequence clustering, and operational taxonomic unit (OTU) delineation were performed as previously described (57). The OTU table was finalized by mapping all unique sequences to the obtained OTUs with the USEARCH (58) global alignment algorithm at a 97% cutoff. A total of 7,968,290 high-quality reads (27,288 ± 6,254 reads per sample) were clustered into 736 OTUs at a threshold of 97% identity.

The sequences of all samples were downsized to 12,300 (1,000 permutations) to standardize sequencing depth. All subsequent analyses were performed on the QIIME platform (version 1.9.1). The alpha diversity of each sample was calculated with observed OTUs, Shannon index, and Faith’s phylogenetic diversity (PD whole tree). Representative sequences for each OTU were built into a phylogenetic tree by FastTree and assigned taxonomic classifications using the SILVA rRNA database project. Bray-Curtis matrices for 16S rRNA gene sequencing samples were computed using QIIME via the beta_diversity script (59). The statistical significance of gut microbiota among different groups was assessed by permutational multivariate analysis of variance (PERMANOVA; 9,999 permutations, *P* < 0.001) in R (3.4.2). PCoA and PERMANOVA were based on the data matrix of the Bray-Curtis distances. To identify the most discriminative OTUs among different groups, sparse partial least-squares discriminant analysis (sPLS-DA) was performed using the mixOmics v6.3.1 (60) R package. Centered log ratio (CLR) transformations were implemented in sPLS-DA to circumvent spurious results. The optimal classification performance of the sPLS-DA model was assessed with the perf function using leave-one-out cross-validation with the smallest error rate.

### Statistical analysis.

Statistical analysis was carried out using Prism version 7 (GraphPad Software, Inc.). One-way analysis of variance (ANOVA) or two-way repeated-measures ANOVA, followed by a Tukey *post hoc* test, was used to analyze variation among the three groups. Differences were considered statistically significant when the *P* value was <0.05.

### Data availability.

Raw Illumina sequence data of the 16S rRNA gene generated in this study are available in the NCBI Sequence Read Archive (SRA) under accession number SRP166705.
